# Supplementary material for: Hybrid Models and Biological Model Reduction with PyDSTool
Source: PLoS Comput Biol. 2012 Aug 9;8(8):e1002628. doi: 10.1371/journal.pcbi.1002628 (PMC3415397; doi:10.1371/journal.pcbi.1002628)
Supplement: Text S4 — Complete source code for the PyDSTool package (version 0.88.120504). Includes API documentation and help files linking to web pages. This file is identical to the current public release on Sourceforge.net. (ZIP) [file pcbi.1002628.s004.zip › PyDSTool/html/PyDSTool.MProject.qt_feature_node-class.html]

xml version="1.0" encoding="ascii"?


PyDSTool.MProject.qt\_feature\_node


| Home | Trees | Indices | Help | PyDSTool |
| --- | --- | --- | --- | --- |

Package PyDSTool ::
Module MProject ::
Class qt\_feature\_node

[hide private]

[frames] | no frames]

# Class qt\_feature\_node

source code

```
object --+        
         |        
   feature --+    
             |    
  feature_node --+
                 |
                qt_feature_node
```

Known Subclasses:
:   - Model.domain\_test
    - , Toolbox.neuro\_data.spike\_feature

---

```
Quantitative feature (regular node).
Add description to note assumptions used for defining feature.

input: a trajectory segment
output: a vector of boolean-valued tolerance tests on the discrepancies between ideal and
  actual features defined by a list of function tests
  e.g. a test returns (residual of ideal-actual) < tolerance
```


|  |  |
| --- | --- |
| Instance Methods | [hide private] |

**Inherited from `feature_node`**:
`__getitem__`,
`__init__`,
`__repr__`,
`__str__`,
`evaluate`,
`propagate_verbosity`,
`set_ref_traj`

**Inherited from `feature_node`** (private):
`_residual_info`

**Inherited from `feature`**:
`__call__`,
`__eq__`,
`__hash__`,
`__ne__`,
`finish`,
`info`,
`postprocess_ref_traj`,
`prepare`,
`reset_metric`,
`validate`

**Inherited from `feature`** (private):
`_find_idx`

**Inherited from `object`**:
`__delattr__`,
`__getattribute__`,
`__new__`,
`__reduce__`,
`__reduce_ex__`,
`__setattr__`


|  |  |
| --- | --- |
| Properties | [hide private] |

**Inherited from `object`**:
`__class__`

| Home | Trees | Indices | Help | PyDSTool |
| --- | --- | --- | --- | --- |

|  |  |
| --- | --- |
| Generated by Epydoc 3.0.1 on Fri May 4 15:24:06 2012 | http://epydoc.sourceforge.net |
